# Reimagining Training During a Pandemic: The Experience of Middle Tennessee GWEP

**DOI:** 10.1093/geroni/igab046.1833

**Published:** 2021-12-17

**Authors:** Shana Rhodes, James Powers

**Affiliations:** 1 Vanderbilt University Medical Center, Nashville, Tennessee, United States; 2 Vanderbilt University, Nashville, Tennessee, United States

## Abstract

A major component of The Middle Tennessee GWEP involves delivery of an annual regional geriatrics update conference. Formerly in-person, the planning committee transformed the 34th Annual Update Conference to a virtual platform within a six-month period. The University partner provided a Zoom platform with licensing and training of program staff. National marketing was achieved through professional societies and purchased e-mailings. Participants numbered 79, including 8 disciplines. Presenters were instructed on platform techniques including screen sharing, polling function, and breakout rooms to enhance audience participation. REDCap registration captured demographic information and facilitated evaluations and post-attendance intention-to-change surveys. Lessons learned were shared with community partners and advisory board members who demonstrated changes in service delivery models and training of new staff to support care to greater numbers of clients and participants. Virtual platforms can extend outreach for valuable learning and service outcomes and maintain high levels of satisfaction among target audiences.

